# Lifelong treatment with atenolol decreases membrane fatty acid unsaturation and oxidative stress in heart and skeletal muscle mitochondria and improves immunity and behavior, without changing mice longevity

**DOI:** 10.1111/acel.12205

**Published:** 2014-02-26

**Authors:** Alexia Gómez, Ines Sánchez-Roman, Jose Gomez, Julia Cruces, Ianire Mate, Mónica Lopez-Torres, Alba Naudi, Manuel Portero-Otin, Reinald Pamplona, Monica De la Fuente, Gustavo Barja

**Affiliations:** 1 Department of Animal Physiology-II, Faculty of Biological Sciences, Complutense University of Madrid (UCM)Madrid, Spain; 2 Department of Experimental Medicine, Faculty of Medicine, University of Lleida-IRBLLEIDALleida, Spain

**Keywords:** aging, atenolol, fatty acid unsaturation, heart rate, longevity, oxidative stress

## Abstract

The membrane fatty acid unsaturation hypothesis of aging and longevity is experimentally tested for the first time in mammals. Lifelong treatment of mice with the β1-blocker atenolol increased the amount of the extracellular-signal-regulated kinase signaling protein and successfully decreased one of the two traits appropriately correlating with animal longevity, the membrane fatty acid unsaturation degree of cardiac and skeletal muscle mitochondria, changing their lipid profile toward that present in much more longer-lived mammals. This was mainly due to decreases in 22:6n-3 and increases in 18:1n-9 fatty acids. The atenolol treatment also lowered visceral adiposity (by 24%), decreased mitochondrial protein oxidative, glycoxidative, and lipoxidative damage in both organs, and lowered oxidative damage in heart mitochondrial DNA. Atenolol also improved various immune (chemotaxis and natural killer activities) and behavioral functions (equilibrium, motor coordination, and muscular vigor). It also totally or partially prevented the aging-related detrimental changes observed in mitochondrial membrane unsaturation, protein oxidative modifications, and immune and behavioral functions, without changing longevity. The controls reached 3.93 years of age, a substantially higher maximum longevity than the best previously described for this strain (3.0 years). Side effects of the drug could have masked a likely lowering of the endogenous aging rate induced by the decrease in membrane fatty acid unsaturation. We conclude that it is atenolol that failed to increase longevity, and likely not the decrease in membrane unsaturation induced by the drug.

## Introduction

Only two known parameters appropriately correlate with animal longevity across species – the rate of mitochondrial reactive oxygen species production (mitROSp) and the membrane fatty acid unsaturation degree. Long-lived mammals and birds show a low rate of mitROS generation (reviewed in Barja, 2013) and have cellular membranes with a low degree of fatty acid unsaturation (Pamplona *et al*., 1996, 2002; Hulbert *et al*., 2007; Naudi *et al*., 2011). These two traits are fundamental for the mitochondrial free radical theory of aging (Harman, 1972; Barja, 2013). The low mitROSp of long-lived species decreases oxidative damage to key molecules such as mtDNA (Barja & Herrero, 2000), and their low membrane fatty acid unsaturation diminishes the intensity of lipid peroxidation and thus membrane and protein damage (Pamplona *et al*., 2002) and the secondary generation of toxic chemicals and free radicals (Pamplona, 2011; Zimniak, 2011).

Nevertheless, correlation does not imply causation. Experimental approaches that can reveal causal connections of those two main traits with aging and longevity are needed. In the case of mitROSp, it is modified by dietary manipulations that increase mean and maximum longevity. Dietary (Gredilla & Barja, 2005), protein (Sanz *et al*., 2004), and methionine restriction (Sanz *et al*., 2006) decrease mitROSp (Sanchez-Román and Barja, 2013) and these three manipulations also increase longevity in rats and mice and improve many health-related parameters (Perrone *et al*., 2013), supporting the idea that the low mitROSp of long-lived animals causally contributes to decreased aging rate in long-lived animal species.

We hypothesize here that experimentally decreasing membrane fatty acid unsaturation can prevent detrimental changes with age and increase longevity in mammals. There are no investigations about the lifelong effect of decreasing tissue membrane fatty acid unsaturation on mammalian longevity and regulation of aging-related parameters. Short-term – 2-week – investigations have shown that atenolol, a β1-adrenergic receptor blocker, given in drinking water, decreases membrane fatty acid unsaturation in the heart of C57BL/6 mice (Sanchez-Roman *et al*., 2010) and rats (Sanchez-Roman *et al*., 2014). The extent of such decrease was almost equal to the difference in unsaturation between mammals differing by one order of magnitude in longevity, such as mice and cows, and it lowered cardiac protein oxidative damage and lipoxidation (Sanchez-Roman *et al*., 2010). This is interesting also because knocked-out mice for the adenylyl cyclase type 5 gene (AC5KO mice), which blocks β-adrenergic receptor signaling and increases the Raf/MEK/ERK signaling pathway, showed increased longevity together with lowered cardiovascular, heart, and bone aging (Yan *et al*., 2007).

Taking advantage of the striking capacity of atenolol to strongly decrease membrane unsaturation in mice, we use it here to experimentally test, for the first time, the hypothesis that lowering the degree of membrane fatty acid unsaturation in a mammal causes beneficial changes during aging and increases longevity. Atenolol has been widely used in humans during the last 3–4 decades to treat various cardiovascular pathologies and has been classically considered a safe drug without significant side effects. A total of 134 mice were treated or not with atenolol in their drinking water during their whole lifespan, and the effect on mean and maximum longevities was studied. At 18 months of age, subgroups of 48 separated ‘pilot’ animals were used to measure cardiovascular and other basic physiological parameters: membrane fatty acid composition and global unsaturation; mitochondrial oxygen consumption and mitROSp; amounts of respiratory complexes (I–IV) and the complex I-related apoptosis-inducing factor (AIF); oxidative damage to mtDNA; oxidative, glycoxidative, and lipoxidative protein modification in heart and skeletal muscle mitochondria; and the amount of phosphorylated extracellular-signaling-regulated kinase (p-ERK) to corroborate activation of the Raf/MEK/ERK signaling pathway by the atenolol treatment. Immunological parameters and various behavioral tests were also performed to better estimate the general functional state of the animals. Although it is generally thought that immune cells have almost exclusively β-2 adrenergic receptors, there are results involving β-1 adrenergic receptor signaling in the modulation of immune responses (Emeny *et al*., 2007), but their immunological relevance has been largely unexplored and the effect of atenolol on the immune cell functions has been scarcely studied. Concerning behavior, a few reports showed decreased anxiety (Deary *et al*., 1991) and antidepressant effects (Al-Tubuly *et al*., 2008) after atenolol treatment, but the impact of atenolol on behavioral parameters has not been investigated. In the case of the cardiovascular parameters, as an increase in mortality was detected only in very old individuals, the measurements were repeated at 35 months of age. A group of young but mature adult control mice of 6 months of age (at sacrifice) maintained in parallel under similar conditions to those of the lifelong control and treated animals was used to check whether atenolol can prevent age-related changes. Heart and skeletal muscle were selected as vital target organs because they are among the ones most affected by the endogenous aging process and are almost fully composed of postmitotic cells.

## Results

### Body and organ weights and physiological parameters

After 16 months of experimentation, the 18-month-old animals [both Old controls and Old atenolol (AT)] had higher total weight and higher heart, kidney, liver, and total visceral fat weight than Young ones, whereas the weights of the spleen, brain, and hind limb skeletal muscle were not modified by aging (results not shown). Visceral fat strongly increased from 0.441 ± 0.051 grams in Young to 2.31 ± 0.20 g in Old controls (424% increase; *P* < 0.00001). The long-term atenolol treatment significantly decreased kidney weight by 7.6% (from 0.655 ±0.016 to 0.605 ± 0.012 g; *P* < 0.01) and visceral adipose tissue mass by 23.8% (from 2.31 ± 0.20 to 1.76 ± 0.18; *P* < 0.02; by a mean of 23.2% when expressed per grams of body mass), whereas total body weight and the weight of the rest of the organs were not affected by the treatment. No differences in food or water intake between Old and Old-AT animals were observed after inspection once every 2 weeks.

The values of various basic physiological parameters are shown in Table S1 (Supporting Information). Old-AT animals had significantly lower routine metabolic rate than Young animals but showed no significant differences compared to Old controls. Neither rectal temperature, heart rate, nor the systolic, mean, and diastolic arterial pressures showed AT- or age-related differences.

### Long-life survival study

**Figure 1:**
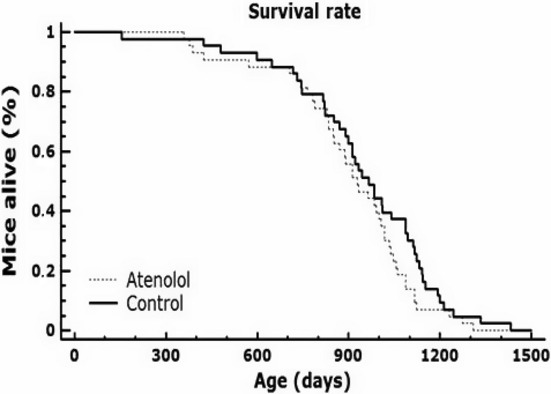
Lifelong survival curves (Kaplan–Meyer survival plots) during the whole lifespan of control and chronically atenolol-treated animals. No significant differences in survival were observed between the two groups with the log-rank and the Wilcoxon tests, while the higher mortality observed with atenolol between 1000 and 1275 days of age was statistically significant (see Table S3). The longest-living control animal reached 3.93 years of age (47,1 months). Eighty-six animals (43 per group) were used in the survival study. Raw data tables of control and atenolol-treated animals are included in the online Supporting Information.

The survival curves of control and AT-treated animals are shown in Fig. 1. No significant differences in mean, median, maximum (90%) longevities, or total curve survival (Tables S2 and S3, Supporting Information) were observed comparing control and AT-treated animals (log-rank and Wilcoxon tests). The longer-lived animal in the control group reached 1433 days of age (3.93 years), and the longer-lived AT mouse reached 1310 days of age (3.6 years). A statistically significant higher mortality in atenolol-treated compared to control animals was observed between 1000 and 1275 days of age (Table S3, Supporting Information). Due to this unexpected finding, the cardiovascular parameters were measured again, more than 1 year after the previous measurements (taken at 18 months of age), in very old animals. These measurements showed that, at 35 months of age, heart rate and systolic, mean, and diastolic blood pressures were significantly lower in Old-AT than in Old controls (Table S4, Supporting Information).

### Mitochondrial oxygen consumption and reactive oxygen species production

Table S5 (Supporting Information) shows the mitochondrial oxygen consumption values. The rate of oxygen consumption of heart mitochondria in state 3 (phosphorylating) with complex I-linked substrates (glutamate/malate) was significantly higher in Old than in Young controls and significantly decreased in Old-AT to values similar to those of Young mice. These differences were not present with succinate + rotenone, a condition in which the inhibitor rotenone avoids electrons to reach complex I by reverse electron flow from complex II. No significant differences due to age or atenolol treatment were observed in heart mitochondria in state 4 (resting) or in skeletal muscle mitochondria with any substrate or mitochondrial state.

The basal rate of ROS production of heart mitochondria was not modified by aging or the atenolol treatment with either glutamate/malate or succinate + rotenone (Table S6, Supporting Information). The maximum rate of heart complex I mitROS production, which was obtained with glutamate + rotenone, was significantly higher in Old controls (but not in Old-AT) than in Young controls. In the case of skeletal muscle mitochondria, no age-related differences in the mitROS generation of control mice were observed with any substrate. The atenolol treatment significantly decreased skeletal muscle maximum complex I mitROS generation (measured with glutamate/malate + rotenone) of Old-AT compared to Old controls. Skeletal muscle mitROS production with glutamate/malate or succinate (which can come from complex I or III) was significantly lower in Old-AT than in Young controls, whereas mitROSp with succinate + rotenone (coming only from complex III) did not change. All these results, taken together, indicate that the decrease in mitROSp induced by atenolol takes place only at complex I.

### Respiratory chain complexes and apoptosis-inducing factor

The amounts of the four respiratory chain protein complexes and AIF are shown in Table S7 (Supporting Information). In the heart, no differences between Old and Young controls were detected for any respiratory complex. The atenolol treatment significantly increased the amount of complex II in heart mitochondria. Heart complex I (30kDa subunit) amount was significantly higher in Old-AT than in Young controls, but no differences were observed between Old-AT and Old controls, and heart complexes III and IV did not show AT-related differences. In skeletal muscle, complexes II and IV decreased and complex III (FeS protein) increased both in Old controls and in Old-AT when compared to Young animals, whereas atenolol-related differences in old animals were not observed for any complex. The amount of AIF protein did not show aging- or AT-related variations either in heart or in skeletal muscle.

### Membrane fatty acid unsaturation

**Figure 2:**
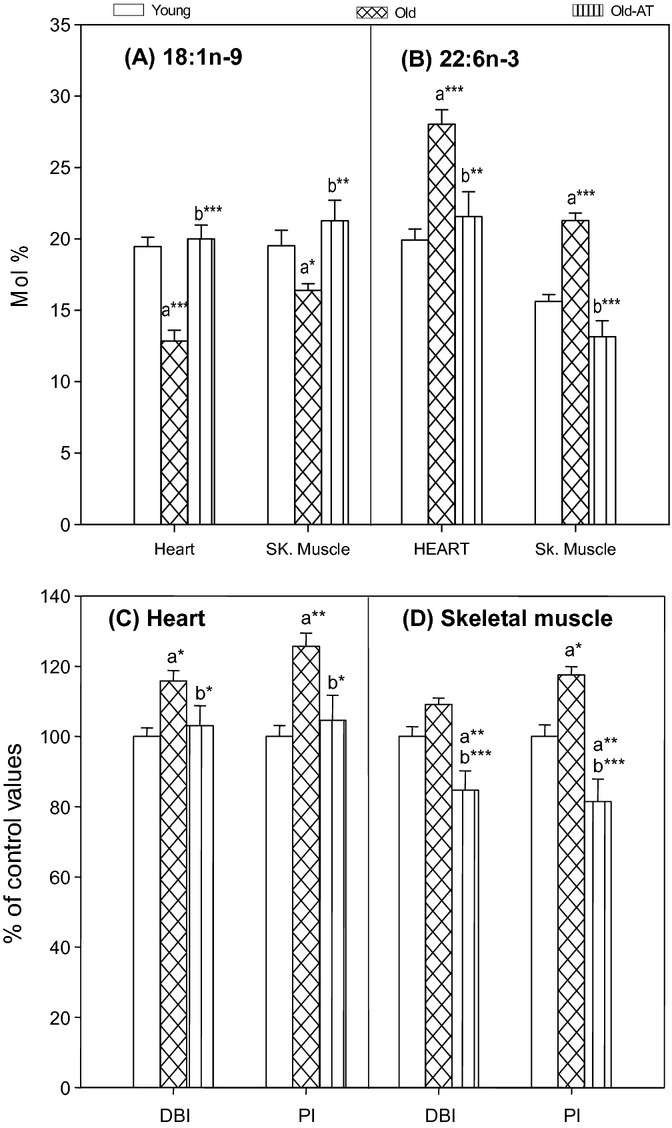
Global fatty acid unsaturation indexes (C, D) and the main fatty acids responsible for their aging- and atenolol-induced modification (A, B: oleic, 18:1n-9; and docosahexaenoic, 22:6n-3 acids) in heart and skeletal muscle mitochondria from Young control, Old control, and Old atenolol-treated mice. Values are means ± SEM from 5 to 6 (heart) or 6 (skeletal muscle) animals and are expressed as absolute values (A, B: mol% of 18:1n-9 and 22:6n-3) or as percentage of those in the Young controls [C, D: double bond index (DBI) and peroxidizability index (PI)]. Control values of unsaturation indexes: 200.9 ± 5.0 (DBI, heart); 213.6 ± 6.7 (PI, heart); 186.9 ± 5.2 (DBI, skeletal muscle); 185.1 ± 6.2 (PI, skeletal muscle). For the calculation of DBI and PI values, see the Materials and Methods section in the online Supporting Information. a*: significantly different from Young controls; b*: significantly different from Old controls; *P < 0.05; **P < 0.01; ***P < 0.001.

The changes induced by aging and AT treatment on fatty acid composition of heart mitochondria are shown in Table S8 (Supporting Information). As a result of these changes, the global indexes of fatty acid unsaturation of heart mitochondria were changed (Fig. 2). Thus, aging increased the heart mitochondria double bond index (DBI) and peroxidizability index (PI), and atenolol decreased the DBI and PI of Old-AT animals to values similar to those of Young controls (Fig. 2C). Quantitatively, the main fatty acids responsible for the change in global unsaturation were 18:1n-9 and especially 22:6n-3. The decrease in 18:1n-9 in Old controls was totally prevented by atenolol, and the increase in 22:6n-3 induced by aging was also totally prevented by the AT treatment (Fig. 2).

The changes induced by aging and AT treatment on fatty acid composition of skeletal muscle mitochondria are shown in Table S9 (Supporting Information). These changes led to aging-related increases in fatty acid unsaturation of skeletal muscle mitochondria (statistically significant for PI) and to AT-related decreases in both DBI and PI (Fig. 2D). In this tissue, the AT-induced decrease was so strong that the DBI and PI decreased in Old-AT to values lower than those of Young controls. Quantitatively, the main fatty acids responsible for the change in global unsaturation were again 18:1n-9 and, especially, 22:6n-3 (Fig. 2). The decrease in 18:1n-9 in Old controls was totally prevented by atenolol (Fig. 2A). The increase in 22:6n-3 induced by aging was totally abolished in Old-AT animals, which showed levels even somewhat below those of Young controls (Fig. 2B).

### Oxidative damage markers

Oxidative damage to heart mtDNA, measured as 8-oxodG (8-oxo-7,8-dihydro-2′deoxyguanosine), tended to increase with aging (nonsignificant difference) and was significantly decreased by the chronic atenolol treatment (Table S10, Supporting Information). In the case of skeletal muscle, nonsignificant trends to higher 8-oxodG values in aged animals were also observed, in this case both in Old control and in Old-AT when compared with Young controls.

**Figure 3:**
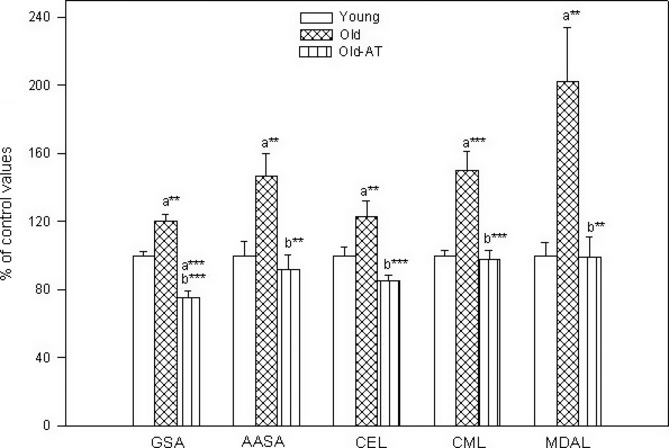
Protein oxidation, glycoxidation, and lipoxidation indicators in heart mitochondria from Young control, Old control, and Old atenolol-treated mice. Values are means ± SEM from six animals and are expressed as percentage of those in the Young controls for each protein modification marker. Control values: 3172 ± 87 (glutamic semialdehyde, GSA); 295 ± 25 AASA, aminoadipic semialdehyde, AASA); 455 ± 22 (carboxyethyl-lysine, CEL); 692 ± 20 (carboxymethyl-lysine, CML); 805 ± 60 (malondialdehyde-lysine, MDAL). Units: μmol/mol lysine. a*: significantly different from Young controls; b*: significantly different from Old controls; **P < 0.01; ***P < 0.001.

The values of the different kinds of protein modification markers (protein oxidation, glycoxidation, and lipoxidation) are shown in Figs S1 and 3. In heart mitochondria, all the five markers measured, glutamic semialdehydes (GSA), aminoadipic semialdehydes (AASA), carboxyethyl-lysine (CEL), carboxymethyl-lysine (CML), and malondialdehyde-lysine (MDAL), were significantly higher in Old than in Young controls (Fig. 3). All these age-related increases were totally prevented by the atenolol treatment, as Old-AT animals always showed lower levels than Old controls and did not show significant differences when compared with Young controls, except for GSA values that were even lower in Old-AT than in Young animals.

In skeletal muscle mitochondria, the five protein markers also showed significantly higher levels in Old than in Young control animals (Fig. S1, Supporting Information). The age-related increases in GSA, AASA, CML, and MDAL were totally prevented by the atenolol treatment because the values in Old-AT were statistically lower than in Old controls and similar to those of Young animals in all cases. Only in the case of CEL did the decrease shown by Old-AT compared to Old controls not reach statistical significance. However, the CEL value of Old-AT animals was statistically similar to that of Young animals.

### Extracellular-signal-regulated kinase signaling proteins

**Figure 4:**
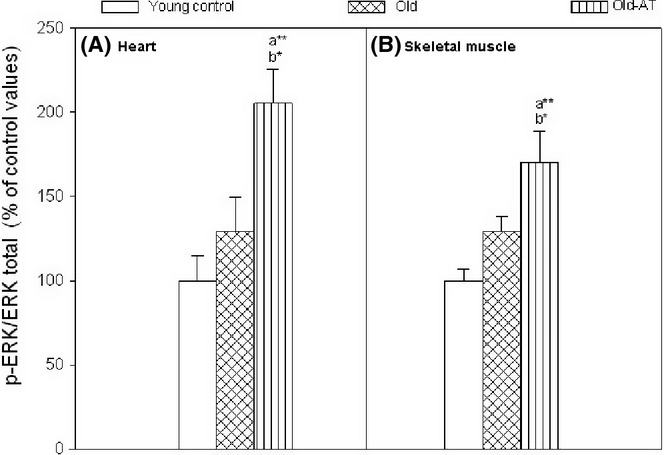
Amounts of extracellular-signal-regulated kinase signaling proteins in heart (A) and skeletal muscle (B) of Young control, Old control, and Old atenolol-treated mice. Values are means ± SEM from 4 animals. Units are ratio of protein content/tubulin. a*: significantly different from Young control; b*: significantly different from Old control; *P < 0.05; ** *P* < 0.01.

The amounts of ERK proteins are shown in Fig. 4. The atenolol treatment significantly increased the amount of p-ERK/total ERK in heart and skeletal muscle of Old-AT mice when compared with Old and Young controls.

### Immune function

**Figure 5:**
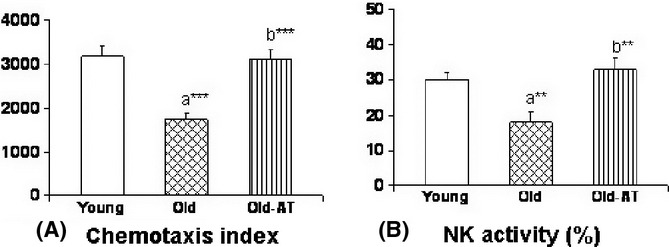
Immune function parameters, chemotaxis (A), and natural killer activity (B), in Young control, Old control, and Old atenolol-treated mice. Values are means ± SEM from 8 animals. a*: significantly different from Young controls; b*: significantly different from Old controls; **P < 0.01; *** *P* < 0.001.

Lymphoproliferation of spleen leukocytes (basal, cpm, and percentage of stimulation in proliferation in response to the mitogens concavalin A and LPS) was measured in Young and Old controls and Old-AT groups, and statistically significant differences between groups were not observed (results not shown). Migration of spleen leukocytes is represented in Fig. 5A as the chemotaxis index. Chemotaxis significantly decreased from Young to Old controls, and this change was totally prevented in Old-AT mice, which showed values similar to those of Young controls. Analogously, natural killer (NK) activity of spleen leukocytes decreased from Young to Old controls, and this change was totally prevented in Old-AT, which showed values statistically similar to those of Young control mice (Fig. 5B).

### Behavior

**Figure 6:**
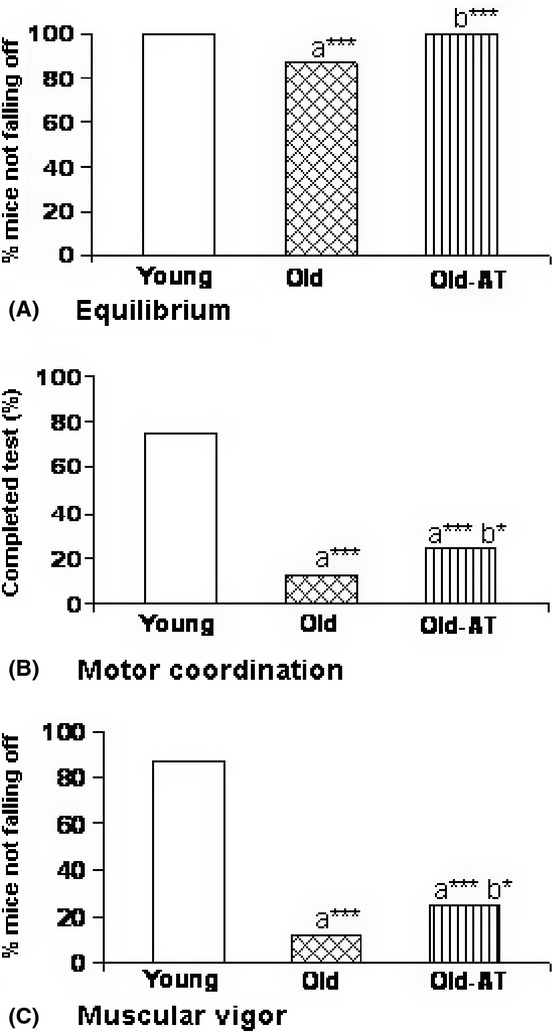
Behavioral sensorimotor abilities in Young control, Old control, and Old atenolol-treated mice. Equilibrium (A) and motor coordination (B) were assayed with the wood rod test, and muscular vigor (C) with the tightrope test. Values are means ± SEM from 8 animals. a*: significantly different from Young controls; b*: significantly different from Old controls; *P < 0.05; *** *P* < 0.001.

In relation to sensorimotor abilities, a significant decrease in equilibrium was observed in Old compared to Young controls, which was totally corrected in Old-AT animals (Fig. 6A). Motor coordination and muscular vigor (Fig. 6B,C) were strongly decreased in Old compared to Young controls. Old-AT mice showed levels of motor coordination and muscular vigor higher than those of Old controls and lower than those of Young controls. Old-AT mice showed significantly higher performance of central rearing in the open-field test and lower defection and urination behavior in the open-field and holeboard tests than Old controls, while no significant differences were detected for horizontal activity, self-grooming behavior, and goal- or non-goal-directed behavior (results not shown).

## Discussion

In this longevity experiment, lifelong treatment of mice with the β1-blocker atenolol lowered one of the two traits appropriately correlating with animal longevity, the membrane fatty acid unsaturation degree of heart and skeletal muscle mitochondria, changing their lipid unsaturation profile toward that present in much longer-lived mammals. The atenolol treatment also lowered visceral adiposity, decreased maximum mitROSp from complex I and oxidative, glycoxidative, and lipoxidative protein damage in both organs, and oxidative damage in heart mtDNA. It also improved chemotaxis and natural killer activities, equilibrium, motor coordination, and muscular vigor and totally or partially prevented the aging-related changes observed for these parameters. However, neither mean, median, nor maximum longevities were modified by the treatment when the whole lifespan was considered. Atenolol increased somewhat the mortality rate only in very old animals, between 1000 and 1275 days of age. This is interesting because this is generally consistent with the results of current meta-analyses in hypertensive human subjects.

In spite of the lowering of oxidative stress and the improvement in immunity and behavior, the general survival and the median, mean, and maximum longevity were not modified by the atenolol treatment. A reason could be the high longevity of the controls of our study, which reached 3.93 years of maximum and 31 and 32 months of mean and median longevity, respectively; these values are higher than the best previously described for the B6D2F1 (C57BL/6xDBA/2N)F_1_ strain: 25.7 months for median longevity and 3.0 years for the maximum longevity of the longest-lived mouse (Yamate *et al*., 1990). The median (32 months) and maximum (47.1 months) longevities of our control mice are similar or higher (maximum longevity) than those of the long-lived mutant AC5KO (C57BL/6Jx129SvJ) mice (33 and 37 months, respectively), which affect the same Raf/MEK/ERK signaling pathway activated by atenolol (Yan *et al*., 2007); therefore, they are also much longer-lived than their corresponding controls, 25 and 33 months, respectively, for the controls of AC5KO. In lifelong survival experiments, increases in longevity are more frequently obtained when the longevity of the controls is on the low range, but rarely when it is nearer to the optimum (see Table 1 in Barja, 2013). The difficulty in increasing the mean lifespan of very long-lived controls is logical because it is difficult to make more ‘squared’ a survival curve that is already very rectangular, and in our case, the shape of the control survival curve (Fig. 1) approaches that situation.

Recently, it was shown that knocking down enzymes of the PUFA biosynthesis pathways in *C. elegans* decreases the DBI and PI due to the same kind of fatty acid differences observed in the interspecies comparative studies (Naudi *et al*., 2011), and in our long-life atenolol study, and that change increases worm longevity (Shmookler Reis *et al*., 2011). This demonstrates the existence of a causal relationship between desaturase/elongase enzymes, membrane unsaturation, and longevity. Another likely reason why longevity was not increased in our AT-treated mice is that atenolol acts far upstream from the final fatty acid changes in the pathway ‘β-receptor-AC-Raf/MEK/ERK signaling to nucleus changes in PUFA biosynthesis pathways – DBI modification’, compared to the *C. elegans* RNAi experiments that directly curtail the expression of these enzymes. The further upstream from the final targets, the more likely is that heavy branching and interconnection during cellular signaling and their metabolic effects will lead to detrimental side effects. Negative side effects of atenolol during most of the study could have been compensated by its multiple positive effects observed in our investigation, leaving global survival finally unchanged. We observed cardiovascular-linked negative effects of atenolol only at the end of the lifespan (between 1000 and 1275 days of age), while other possible negative effects at younger ages could have been unnoticed due to the particular selection of measured parameters. Finally, it is generally agreed that many traits rather than a single one must simultaneously and coordinately change in the right direction in order for (maximum) longevity to substantially increase (Barja, 2008). This can also help to explain the lack of lifespan extension observed in the present investigation.

The lack of changes in longevity with atenolol treatment is in line with almost all previous studies attempting to retard aging using drugs and other small molecules. Thus, neither aspirin, antidiabetics including metformin, resveratrol, and melatonin, nor many other drugs have consistently demonstrated increases in rodent maximum longevity (reviewed in Spindler, 2012). Perhaps, the only exception to this could be rapamycin, which led to modest but consistent 4–14% increases in maximum longevity (90th percentile survival age) after parallel replication of the longevity experiment at three institutions (Harrison *et al*., 2009). In contrast, many different single-gene mutant mice have shown up to 40% increases in maximum longevity. Detrimental side effects of the drugs can be responsible for such difference with the outcome of the gene-modifying investigations. Perhaps, an analogous approach to that used in *C. elegans* could increase longevity in mammals by more directly down-regulating PUFA biosynthesis enzymes in mice. Previous studies disrupting the FADS2 gene coding for delta-6 desaturase have resulted in −/− viable but sterile mice suffering various pathologies, including ulcerative dermatitis, splenomegaly, and intestinal ulceration (Stoffel *et al*., 2008; Stroud *et al*., 2009). Perhaps, alternative approaches leading to strong but not total depletion of the enzymes responsible for synthesizing highly unsaturated PUFA could help to design useful tests of the unsaturation-longevity hypothesis in mammals (Pamplona *et al*., 1996, 2002).

Interestingly, while writing this manuscript, we were aware that a very similar study had recently been published (Spindler *et al*., 2013). In that study, 36 B6C3F1 male mice received continuously until death other two β1-blockers, metoprolol and nebivolol mixed in the diet, beginning the treatment at 12 months of age. The final effect on survival of these two drugs was approximately 10% and 6.4% increases in median lifespan, respectively, with no increase in maximum lifespan. Visual inspection of the survival curves on that study (Fig. S1a,b) suggests to us that the maximum lifespan of the β1-blocker-treated animals tended (not significantly) to be somewhat smaller than that of their controls. The maximum longevity in that study (around 1400 days according to the figure survival plots) was very near to that obtained in our investigation (1433 days), and median lifespan was also very similar in both studies, 983 days in the Spindler *et al*. (2013) study and 967 days in our investigation. While we consider the results of both studies very similar, the differences concerning median lifespan and survival could be due to the various differences in design: use of different mice strains, use of different β1-blockers, and different doses and ways of administration. In addition, the drug treatments were started at 12 months of age in the Spindler *et al*. (2013) study and at 2 months of age in our case. Most interestingly, Spindler *et al*. (2013) found significantly lower body weight and higher mortality with metoprolol and nebivolol at 1245–1250 days of age and later and suggested that this was due to mild drug toxicity in very old mice due to liver aging. This result is strikingly similar to our detection of a significantly higher mortality with atenolol between 1000 and 1275 days of age, which would also agree with current meta-analyses from hypertensive human patients (see below). In our opinion, the general similarity in the outcome of both studies increases their reliability, as it means that their results have been generally replicated independently at two sites in spite of some differences in design.

It is known that atenolol increases human survival after acute myocardial infarction and prevents premature death in patients with coronary heart diseases and systolic heart failure. However, meta-analyses are currently raising the question whether β-blockers (atenolol is the one most frequently used) are appropriate for the treatment of hypertension (Lindholm *et al*., 2005; Cockcroft & Pedersen, 2012). Compared with other antihypertensive drugs, β-blockers showed higher all-cause mortality, cardiovascular mortality, myocardial infarction, and heart failure, especially in association with a lower heart rate. Concerning aged subjects, the treatment of hypertension with β-blockers has been questioned in elderly patients (Lindholm *et al*., 2005), and there is little information on the possible different effects of atenolol as a function of age. The negative effects of atenolol can be due to its lowering heart rate, ineffective lowering or even increase in central aortic pressure, lowered diastolic pressure, or negative metabolic effects on glucose and lipid metabolism (Bangalore *et al*., 2008). In patients with slower heart rate, the reflected wave from the periphery reaches the next wave in systole (instead of in diastole) and hence may increase central aortic pressure.

When the survival statistical analysis was restricted to the time window between 1000 and 1275 days of age, atenolol showed a significant increase in mortality rate. This shows that atenolol can increase mortality of a mammal in very old but not in young or middle-aged individuals, which would be in line with the meta-analyses in human patients, especially because hypertension is common in old individuals. This is especially interesting because human hypertensive patients are usually not medicated until midlife diagnosis but still seem to show an increased atenolol-induced mortality. We found that 8–13% decreases in arterial pressures (mean, systolic, and diastolic) and especially in heart rate (by 20%) occur at the same time window at which mortality increases in the atenolol group, in very old (35 months old) animals, but not in young or moderately (18 months) old mice in which survival is indistinguishable from that of controls. This agrees with the main change associated with the negative effects of atenolol on human hypertensive patients, a decrease in heart rate (Bangalore *et al*., 2008). Atenolol-induced decreases in heart rate and cardiac output and transitory peaks of variation of arterial pressure due to poorer pressure regulation in very old animals, which have a myocardium heavily degenerated by aging-related damage and the main systemic arteries stiffened by atherosclerosis, can lead to heart failure and be fatal. Decreased arterial elasticity would amplify drops in blood flow to critical main organs including the heart and brain when arterial pressure variations occur in the downward direction. Such processes are absent (heart rate and arterial pressures are normal at 18 months of age) or are better tolerated in younger animals. They could be among the side effects of the drug counteracting the beneficial effects on oxidative stress, immune function, and behavior found in this long-life study and help to explain why longevity was not changed in spite of those benefits.

The chronic atenolol treatment increased the amount of p-ERK, corroborating activation of the Raf/MEK/ERK signaling pathway, as in long-lived mutant AC5KO mice (Yan *et al*., 2007). Neither body weight, routine metabolic rate, rectal temperature, cardiovascular parameters, nor mitochondrial oxygen consumption was changed by atenolol in 18-month-old animals, except for an increase in heart mitochondrial oxygen consumption with glutamate/malate in state 3 (phosphorylating) in the Old control group, which was prevented by atenolol. This suggests that, at least until 18 months of age, the treatment did not promote overt negative side effects. The lack of modification of total body and organ weights (except for a decrease in kidney weight) and the absence of detection of variations in food intake indicate that the many observed beneficial effects of atenolol are not due to caloric restriction.

Increases in visceral fat in old humans are associated with degenerative illnesses, including type 2 diabetes, metabolic syndrome, and atherosclerosis. Avoiding such increases is considered healthy. In our study, atenolol provided partial but substantial protection against increases in visceral fat. The strong age-related increase in visceral fat from Young to Old controls (424% increase) was 29.5% smaller from Young controls to Old-AT (299% increase) and 32% smaller when expressed as grams of fat per unit body mass. The strong decrease in visceral adiposity induced by atenolol in Old animals (23.8% decrease) is also striking because long-term nutritional interventions extending lifespan in rodents, such as 40% dietary restriction and 80% methionine restriction (Perrone *et al*., 2013), also strongly decrease visceral adiposity per gram of body weight (Malloy *et al*., 2006) while they induce strong decreases in growth and final body size. In our case, however, the lowered visceral adiposity was not accompanied by any detrimental decreases in body size, similar to what happens in 40% methionine restriction (Sanchez-Roman & Barja, 2013), although the effect of this milder intervention on longevity has not been investigated.

The AT treatment did not modify the rate of mitROSp of heart mitochondria, except for avoidance of an age-related increase in maximum complex I ROS generation. In the case of skeletal muscle, no age-related differences in mitROS generation were observed, and atenolol treatment decreased complex I mitROSp in Old mice. This is the respiratory complex that shows low or decreased mitROS generation in long-lived species or in caloric-restricted life-extended animals (Barja, 2013). However, none of the variations in mitROSp observed in either organ are due to changes in the amount of complex I as revealed by the Western blots of the respiratory complexes. Therefore, they must be due to qualitative complex I modifications such as those leading to a decrease in the reduction state and/or the reducing midpoint potential of the complex I ROS generator/s. In contrast, mtROSp lowering through decreases in complex I content has been described in dietary or methionine-restricted animals (Sanchez-Roman & Barja, 2013), although qualitative complex I changes also seem to be involved in these cases.

There is a negative relationship between the tissue and mitochondrial degree of membrane fatty acid unsaturation and longevity in all the animal species studied to date (reviewed in Pamplona *et al*., 2002; Hulbert *et al*., 2007; Naudi *et al*., 2011). This makes the membranes of long-lived animals highly resistant to lipid peroxidation, because the sensitivity of fatty acids to lipid peroxidation increases exponentially as a function of the number of double bonds per molecule. In our study, the atenolol treatment successfully and significantly decreased the global fatty acid mitochondrial membrane unsaturation measured as the DBI in heart and skeletal muscle (11% and 22.4% decreases, respectively) and as the PI also in both tissues (16.8% and 30.8% decreases). These results agree with our short-term study in total heart tissue of C57BL/6 mice (Sanchez-Roman *et al*., 2010). Concerning the intensity of the obtained effect, for example, in skeletal muscle, atenolol decreased the PI of Old-AT mice to a value similar to that of mammals with a fivefold higher longevity (14.7 years) according to data in Fig. 7A from Hulbert *et al*. (2007). Interestingly, the DBI and PI significantly increased with aging from Young to Old controls (nonsignificant trend for heart DBI), whereas the decrease induced by atenolol brought back the DBI and PI to values similar to (heart) or even lower (skeletal muscle) than those of Young mice. Thus, the atenolol treatment was able to fully prevent all the age-related changes in mitochondrial membrane unsaturation. The decreases in DBI and PI induced by atenolol were mainly due to decreases in the most highly unsaturated fatty acid, the PUFA docosahexaenoic acid (22:6n-3), aided by increases in the MUFA oleic acid (18:1n-9) in both tissues. Docosahexaenoic acid (22:6n-3) has six double bonds and consequently has five bis-allylic hydrogens per chain. It is 320 times more susceptible to ROS attack than oleic acid (18:1n-9; Holman, 1954; Hulbert *et al*., 2007).

It is expected that changes in membrane unsaturation would affect not only the membrane lipids and proteins but also the levels of modified proteins in cells in general and the mtDNA, which is situated near or perhaps even in contact with the very abundant inner mitochondrial membranes. In agreement with that, the five markers of protein oxidation, glycoxidation, and lipoxidation measured increased during aging, and all these changes (except for CEL in skeletal muscle) were totally prevented in Old-AT mice back to the levels shown by Young animals. It is generally accepted that aging increases protein oxidative modification, whereas experimental procedures that extend longevity, such as dietary (Barja, 2013) and methionine restriction (Sanchez-Roman & Barja, 2013), decrease it. Notably, the strongest age-related changes were observed for the lipoxidation marker MDAL, which is in agreement with our previous studies using other longevity-related models.

Concerning mtDNA, in previous short-term studies we have shown that effectively increasing tissue fatty acid unsaturation by feeding the animals with the highly unsaturated (n-3) menhaden oil, compared to diets prepared with saturated fats, increased CML and MDAL in proteins and notably the 8-oxodG level in mtDNA (and not in the nuclear DNA) of rat kidney and brain (Pamplona *et al*., 2004). Nonsignificant trends to 8-oxodG increases in mtDNA with aging (especially strong in skeletal muscle) and a significant decrease in 8-oxodG in the heart of Old-AT mice to values similar to those of Young mice were observed. This agrees with current models indicating that not only mitROSp, but the membrane unsaturation degree, can contribute to mtDNA oxidative damage during aging, likely due to secondary free radicals released by lipid peroxidation of the very abundant mitochondrial membranes (Barja, 2013). These are situated very near to the sites of mitROSp and to mtDNA itself, while lipids are scarce in the nucleus, avoiding the generation of lipid peroxidation products in the vicinity of nuclear DNA.

Age and atenolol treatment also affected important physiological functions, such as immunity and behavior, similarly to the changes observed for membrane fatty acid unsaturation and oxidative damage markers. The capacity for chemotaxis, which allows the migration of leukocytes toward the inflammatory focus, constitutes one of the first steps of the immune response, and NK activity is the first line of immune defense against viral infections and neoplasia. Previous studies in CD1 and BALB/c mice showed that the chemotactic response, the lymphoproliferative response, and the NK activity were profoundly and negatively affected by aging in leukocytes from different locations, such as the peritoneum, thymus, lymphatic nodes, and spleen (De la Fuente *et al*., 2004; De la Fuente, 2008). In agreement with those studies, in the present investigation chemotaxis and NK activity were lower in old animals than in young ones. These age-related decreases were prevented by atenolol in Old-AT mice. Atenolol increases various activities of immune cells *in vitro* (Guo *et al*., 2011), but this had not been described previously *in vivo*.

The effects of atenolol on the behavioral capacities of mice were assessed through a wide battery of behavioral tests. Previous studies have documented age-related impairments in sensorimotor abilities, motor coordination, and muscular vigor in ICR-CD1 mice (Baeza *et al*., 2010). In our lifelong study, aging decreased equilibrium, motor coordination, and muscular vigor in Old compared to Young controls, and all these detrimental changes were totally (equilibrium) or partially prevented by atenolol. In addition, atenolol increased the percentage of Old mice that performed central rearing in the open-field test. Rearing is considered the best parameter to estimate non-goal-directed vertical exploratory activity (Escorihuela *et al*., 1999) and indicates the interest or ability of the animal to interact with the environment. Increases in defecation and urine incontinence also occur in old rodents. The observed decrease in defecation and urination in Old-AT mice compared to Old controls indicates a lower anxiety status in these animals. These results agree with the decreases in anxiety (Deary *et al*., 1991) and depression (Al-Tubuly *et al*., 2008) reported after treatment with atenolol in human patients.

In summary, in this study long-term treatment with atenolol effectively decreased membrane fatty acid unsaturation in mice toward values typical of much longer-lived animals, lowering visceral adiposity, decreasing many markers of oxidative stress, and improving immune and behavioral functions during aging without changing longevity. We conclude that it is atenolol that failed to increase longevity, and likely not the decrease in membrane unsaturation.

## Experimental procedures

### Animals and study design

A total of 134 B6D2F1 (C57BL/6 female × DBA/2 male**)** male mice were maintained under SPF conditions receiving *ad libitum* a standard rodent chow (Panlab, Spain) in individual mouse cages during their whole lifespan. Eighty-six of these animals (43 Old control and 43 Old-AT-treated) were used only to obtain the survival curves. The atenolol treatment started at 2 months of age. Forty-eight separated ‘pilot’ animals were used to measure the different physiological and biochemical parameters at 16 months of age after sacrifice by cervical dislocation. A young control group (6 months of age) was also included. Atenolol (Sigma, A7655) was given in drinking water at 0.1 g L^−1^, which resulted in a mean atenolol intake of 0.559 mg per mouse per day. Just after cervical dislocation, tissues were immediately processed to isolate functional mitochondria for respiration and mitROS generation measurements. Tissue and excess mitochondrial samples were stored at −80 °C for the posterior analyses. Detailed methods of the physiological parameters and the measurements in functional mitochondria referred to below are described in the online Supporting Information.

### Physiological parameters

Rectal temperature was measured using a rectal probe, the routine metabolic rate was measured by closed system respirometry, and heart rate and blood pressures were measured with a tail-cuff manometer.

### Measurements in functional mitochondria

Heart and skeletal muscle mitochondria were obtained at 4 °C from fresh tissue by differential centrifugation essentially as described in Sanz *et al*. (2006). The rate of mitochondrial oxygen consumption was measured at 37 °C with a Clark-type O_2_ electrode in the absence (state 4 resting) and in the presence (state 3 phosphorylating) of 500 μM ADP. The rate of mitochondrial ROS production (O_2_^−^ + H_2_O_2_, as excess SOD was added) was assayed by measuring the increase in fluorescence due to oxidation of homovanillic acid by H_2_O_2_ in the presence of horseradish peroxidase (Sanz *et al*., 2006).

### Measurement of mitochondrial complexes I to IV, apoptosis-inducing factor, and extracellular-signal-regulated kinase

The amounts of the mitochondrial respiratory chain complexes (I–IV), the complex I regulatory factor AIF, and ERK1/2 and phospho-ERK1/2 were estimated using Western blot analyses. Detailed methods are described in the online Supporting Information.

### Fatty acid analyses, their biosynthetic enzymes, and global fatty acid unsaturation indexes

Fatty acids from mitochondrial lipids were analyzed as methyl ester derivatives by gas chromatography (GC) as previously described (Sanchez-Roman *et al*., 2010). Results are expressed as mol%. Calculation of the density of double bonds in the mitochondrial membranes (double bond index, DBI; and peroxidizability index, PI) is described in the online Supporting Information.

### Oxidative damage to mtDNA (8-oxodG)

After isolation, mtDNA was digested to deoxynucleoside level. Steady-state oxidative damage to mtDNA was estimated by measuring the level of 8-oxodG referred to that of the nonoxidized base (deoxyguanosine, dG). 8-OxodG and dG were analyzed by HPLC with online electrochemical and ultraviolet detection.

### Oxidation-derived protein damage markers

Glutamic semialdehydes, AASA, CML, CEL, and MDAL in heart and skeletal muscle mitochondrial proteins were determined by GC/MS with selected ion monitoring. Detailed methods are described in the online Supporting Information.

### Immune function parameters

The number of leukocytes was determined in cell suspensions obtained from the spleen. Cellular viability was higher than 95% in all cases. Lymphoproliferation, chemotaxis (directed migration), and natural killer (NK) activity assays were performed as referred in the online Supporting Information.

### Behavioral tests

The open-field and holeboard tests were carried out to analyze exploratory and anxiety-like behaviors. Behavioral tests were performed as referred in the online Supporting Information.

### Statistical methods

Comparisons between the three groups of animals were performed by one-way ANOVA. Comparison of long-life survival of Old control and Old-AT-treated animals was analyzed with the log-rank and Wilcoxon tests. *P* < 0.05 was selected as the minimum level of statistical significance.

## Abbreviations

AASA: aminoadipic semialdehydes
AIF: apoptosis-inducing factor
AT: atenolol
CEL: carboxyethyl-lysine
CML: carboxymethyl-lysine
DBI: double bond index
GSA: glutamic semialdehydes
MDAL: malondialdehyde-lysine
mitROS: mitochondrial reactive oxygen species
mtDNA: mitochondrial DNA
p-ERK: phosphorylated extracellular-signal-regulated kinase
PI: peroxidizability index
8-oxodG: 8-oxo-7,8-dihydro-2′deoxyguanosine

## Supporting Information

**Fig. S1** Protein oxidation, glycoxidation and lipoxidation indicators in skeletal muscle mitochondria from Young control, Old control and Old atenolol treated mice. **Table S1** Basic physiological parameters in Young control, Old* control, and Old* atenolol treated mice at 16-18 months of age. **Table S2** Mean, median and maximum longevities in control and life-long atenolol-treated mice. **Table S3** Statistical and analysis of total and partial survival curves in control and life-long atenolol-treated mice. **Table S4** Heart rate and blood pressures in Old control and Old atenolol treated mice at 35 months of age. **Table S5** Mitochondrial oxygen consumption in Young control, Old control, and Old atenolol treated mice. **Table S6** Mitochondrial ROS production in Young control, Old control, and Old atenolol treated mice. **Table S7** Amounts of respiratory complexes and AIF in mitochondria from Young control, Old control, and Old atenolol treated mice. **Table S8** Fatty acid composition (mol%) of heart mitochondrial lipids of Young control, Old control, and Old atenolol treated mice. **Table S9** Fatty acid composition (mol%) of skeletal muscle mitochondrial lipids of Young control, Old control, and Old atenolol treated mice. **Table S10** Oxidative damage in mitochondrial DNA in Young control, Old control, and Old atenolol treated mice.

## References

[b1] Al-Tubuly RA, Aburawi SM, Alghzewi EA, Gorash ZM, Errwami S (2008). The effect of sympathetic antagonists on the antidepressant action of alprazolam. Libyan J. Med.

[b2] Baeza I, De Castro NM, Gimenez-Llort L, De la Fuente M (2010). Ovariectomy, a model of menopause in rodents, causes a premature aging of the nervous and immune systems. J. Neuroimmunol.

[b3] Bangalore S, Sawhney S, Messerli FH (2008). Relation of beta-blocker-induced heart rate lowering and cardioprotection in hypertension. J. Am. Coll. Cardiol.

[b4] Barja G (2008). The gene cluster hypothesis of aging and longevity. Biogerontology.

[b5] Barja G (2013). Updating the mitochondrial free radical theory of aging: an integrated view, key aspects and confounding concepts. Antioxid. Redox Signal.

[b6] Barja G, Herrero A (2000). Oxidative damage to mitochondrial DNA is inversely related to maximum life span in the heart and brain of mammals. FASEB J.

[b7] Cockcroft JR, Pedersen ME (2012). β-Blockade: benefits beyond blood pressure reduction?. J. Clin. Hypertens.

[b8] De la Fuente M (2008). Role of neuroimmunomodulation in aging. NeuroImmunoModulation.

[b9] De la Fuente M, Baeza I, Guayerbas N, Puerto M, Castillo C, Salazar V, Ariznavarreta C, Tresguerres JA (2004). Changes with aging in several leukocyte functions of male and female rats. Biogerontology.

[b10] Deary IJ, Capewell S, Hajducka C, Muir AL (1991). The effects of captopril vs atenolol on memory, information processing and mood: a double-blind crossover study. Br. J. Clin. Pharmacol.

[b11] Emeny RT, Gao D, Lawrence DA (2007). Beta1-adrenergic receptors on immune cells impair innate defenses against Listeria. J. Immunol.

[b12] Escorihuela RM, Fernández-Teruel A, Gil L, Aguilar R, Tobeña A, Driscoll P (1999). Inbred Roman high- and low-avoidance rats: differences in anxiety, novelty-seeking, and shuttlebox behaviors. Physiol. Behav.

[b13] Gredilla R, Barja G (2005). Minireview: the role of oxidative stress in relation to caloric restriction and longevity. Endocrinology.

[b14] Guo YP, Liu Y, Li JB, Huang Y, Qi HP, Xie J, Cui XG, Yue ZY, Li WZ (2011). Chronic beta-adrenoreceptor antagonists upregulate the rat alveolar macrophage adrenergic system through the beta1-subtype. Cell. Physiol. Biochem.

[b15] Harman D (1972). The biological clock: the mitochondria?. J. Am. Geriatr. Soc.

[b16] Harrison DE, Strong R, Sharp ZD, Nelson JF, Astle CM, Flurkey K, Nadon NL, Wilkinson JE, Frenkel K, Carter CS, Pahor M, Javors MA, Fernandez E, Miller RA (2009). Rapamycin fed late in life extends lifespan in genetically heterogeneous mice. Nature.

[b17] Holman RT, Holman RT, Lundberg WO, Malkin T (1954). Autoxidation of fats and related substances. Progress in Chemistry of Fats and Other Lipids.

[b18] Hulbert T, Pamplona R, Buffenstein R, Buttemer WA (2007). Life and death: metabolic rate, membrane composition and lifespan of animals. Physiol. Rev.

[b19] Lindholm LH, Carlberg B, Samuelson O (2005). Should β-blockers remain the first choice in the treatment of primary hypertension? A meta-analysis. Lancet.

[b20] Malloy VL, Krajcik RA, Bailey SJ, Hristopoulos G, Plummer JD, Orentreich N (2006). Methionine restriction decreases visceral fat mass and preserves insulin action in aging male Fischer 344 rats independent of energy restriction. Aging Cell.

[b21] Naudi A, Jove M, Ayala V, Portero-Otín M, Barja G, Pamplona R (2011). Regulation of membrane unsaturation as antioxidant adaptive mechanisms in long-lived animal species. Free Rad. Antioxid.

[b22] Pamplona R (2011). Advanced lipoxidation end-products. Chem. Biol. Interact.

[b23] Pamplona R, Prat J, Cadenas S, Rojas C, Pérez-Campo R, López-Torres M, Barja G (1996). Low fatty acid unsaturation protects against lipid peroxidation in liver mitochondria from longevous species: the pigeon and human case. Mech. Ageing Dev.

[b24] Pamplona R, Barja G, Portero-Otín M (2002). Membrane fatty acid unsaturation, protection against oxidative stress, and maximum life span: a homeoviscous-longevity adaptation. Ann. N. Y. Acad. Sci.

[b25] Pamplona R, Portero-Otín M, Sanz A, Requena J, Barja G (2004). Modification of the longevity-related degree of fatty acid unsaturation modulates oxidative damage to proteins and mitochondrial DNA in liver and brain. Exp. Gerontol.

[b26] Perrone CE, Malloy VL, Orentreich DS, Orentreich N (2013). Metabolic adaptations to methionine restriction that benefit health and lifespan in rodents. Exp. Gerontol.

[b27] Sanchez-Roman I, Barja G (2013). Regulation of longevity and oxidative stress by nutritional interventions: role of methionine restriction. Exp. Gerontol.

[b28] Sanchez-Roman I, Gomez J, Naudi A, Ayala V, Portero-Otín M, Lopez-Torres M, Pamplona R, Barja G (2010). The β-blocker atenolol lowers the longevity-related degree of fatty acid unsaturation, decreases protein oxidative damage and increases ERK signaling in the heart of C57BL/6 mice. Rejuvenation Res.

[b29] Sanchez-Roman I, Gomez A, Naudí A, Jove M, Gómez J, Lopez-Torres M, Pamplona R, Barja G (2014). Independent and additive effects of atenolol and methionine restriction on lowering rat heart mitochondria oxidative stress. J. Bioenerg. Biomembr.

[b30] Sanz A, Caro P, Barja G (2004). Protein restriction without strong caloric restriction decreases mitochondrial oxygen radical production and oxidative DNA damage in rat liver. J. Bioenerg. Biomembr.

[b31] Sanz A, Caro P, Ayala V, Portero-Otin M, Pamplona R, Barja G (2006). Methionine restriction decreases mitochondrial oxygen radical generation and leak as well as oxidative damage to mitochondrial DNA and proteins. FASEB J.

[b32] Shmookler Reis RJ, Xu L, Lee H, Chae M, Thaden JJ, Bharill P, Tazearslan C, Siegel E, Alla R, Zimniak P, Ayyadevara S (2011). Modulation of lipid biosynthesis contributes to stress resistance and longevity of *C. elegans* mutants. Aging (Albany NY).

[b33] Spindler SR (2012). Review of the literature and suggestions for the design of rodent survival studies for the identification of compounds that increase health and life span. Age (Dordr).

[b34] Spindler SR, Mote PL, Li R, Dhahbi JM, Yamawaka A, Flegal JM, Jeske DR, Li R, Lublin AL (2013). β-1-Adrenergic receptor blockade extends the life span of Drosophila and long-lived mice. Age (Dordr).

[b35] Stoffel W, Holz B, Jenke B, Binczek E, Günter RH, Kiss C, Karakesisoglou I, Thevis M, Weber AA, Arnhold S, Addicks K (2008). Delta-6 desaturase (FADS2) deficiency unveils the role of omega-3 and omega-6 polyunsaturated fatty acids. EMBO J.

[b36] Stroud CK, Nara TY, Roqueta-Rivera M, Radlowski EC, Lawrence P, Zhang Y, Cho BH, Segre M, Hess RA, Brenna JT, Haschek WM, Nakamura MT (2009). Disruption of FADS2 gene in mice impairs male reproduction and causes dermal and intestinal ulceration. J. Lipid Res.

[b37] Yamate J, Tajima M, Kudow S, Sannai S (1990). Background pathology in BDF_1_ mice allowed to live out their life-span. Lab. Anim.

[b38] Yan L, Vatner DE, O’Connor JP, Ivessa A, Ge H, Chen W, Hirotani S, Ishikawa Y, Sadoshima J, Vatner SF (2007). Type 5 adenylyl cyclase disruption increases longevity and protects against stress. Cell.

[b39] Zimniak P (2011). Relationship of electrophilic stress to aging. Free Radic. Biol. Med.

