# Supplementary material for: Lifelong treatment with atenolol decreases membrane fatty acid unsaturation and oxidative stress in heart and skeletal muscle mitochondria and improves immunity and behavior, without changing mice longevity
Source: Aging Cell. 2014 Feb 26;13(3):551–60. doi: 10.1111/acel.12205 (PMC4326892; doi:10.1111/acel.12205)
Supplement: Supplementary file 1 — Fig. S1 Protein oxidation, glycoxidation and lipoxidation indicators in skeletal muscle mitochondria from Young control, Old control and Old atenolol treated mice. Table S1 Basic physiological parameters in Young control, Old* control, and Old* atenolol treated mice at 16-18 months of age. Table S2 Mean, median and maximum longevities in control and life-long atenolol-treated mice. Table S3 Statistical and analysis of total and partial survival curves in control and life-long atenolol-treated mice. Table S4 Heart rate and blood pressures in Old control and Old atenolol treated mice at 35 months of age. Table S5 Mitochondrial oxygen consumption in Young control, Old control, and Old atenolol treated mice. Table S6 Mitochondrial ROS production in Young control, Old control, and Old atenolol treated mice. Table S7 Amounts of respiratory complexes and AIF in mitochondria from Young control, Old control, and Old atenolol treated mice. Table S8 Fatty acid composition (mol%) of heart mitochondrial lipids of Young control, Old control, and Old atenolol treated mice. Table S9 Fatty acid composition (mol%) of skeletal muscle mitochondrial lipids of Young control, Old control, and Old atenolol treated mice. Table S10 Oxidative damage in mitochondrial DNA in Young control, Old control, and Old atenolol treated mice. [file acel0013-0551-sd1.doc]

**SUPPORTING INFORMATION**

**Life-long treatment with atenolol decreases membrane fatty acid unsaturation and oxidative stress in heart and skeletal muscle mitochondria and improves immunity and behaviour without changing mice longevity**

Alexia Gómez,^1^ Ines Sánchez-Roman,^1^ Jose Gomez,^1^ Julia Cruces^1^, Ianire Mate^1^, Mónica Lopez-Torres,^1^ Alba Naudi,^2^ Manuel Portero-Otin^2^ Reinald Pamplona^2^, M. De la Fuente^1^ and Gustavo Barja^1^

**^1^**Department of Animal Physiology-II, Faculty of Biological Sciences, Complutense University of Madrid (UCM), Spain

^2^Department of Experimental Medicine, Faculty of Medicine, University of Lleida-IRBLLEIDA, Lleida, Spain

**Table 1S. Basic physiological parameters in Young control, Old* control, and Old* atenolol treated mice at 16-18 months of age**

Young Old Old-AT

Routine metabolic rate 4.18±0.33 3.54±0.28 3.15±0.30^a*^

(ml O_2_/min . g)

Rectal temperature (ºC) 35.86±0.48 35.35±0.15 35.7±0.25

Heart rate (beats/min) 624.6±31.1 642.4±16.9 629.8±16.6

Arterial blood pressure (Systolic) 235.1±11.5 225.2±4.3 232.1±2.7

Arterial blood pressure (Mean) 215.8±11.1 199.1±6.8 209.7±2.9

Arterial blood pressure (Diastolic) 206.5±11.1 186.5±9.3 199.0±3.6

Values are means ± SEM from 8 different animals. Old animals had 16-18 months of age at the time of measurement. Blood pressure units: mm of Hg. a* = significantly different from Young control; * P<0.05.

**Table 2S. Mean, median and maximum longevities in control and life-long atenolol-treated mice**

Control AT Log Rank test Wilcoxon test (P<) (P<)

Mean survival time^a*^ 944±37 901±34 0.74 0.73

(mean longevity)

Median survival time 967 928 0.73 0.68

Maximum survival (90%)^b*^ 1,140 1,116 0.16 0.30

Maximum longevity 1,433 1,310

(of the last animal alive) (3.93 years) (3.6 years)

Survival times and longevities are expressed in days (± SEM in the case on mean longevity), come from the 86 animals (43 controls and 43 AT animals) on the survival study, and correspond to the survival curves in Fig. 6. a*: statistical Log Rank and Wilcoxon tests for 50% survival, until 716 days of age. The t-student test for mean longevity was not significant either (P<0.19); b* the maximum survival (90%) shown in the table corresponds to the “age when 90% of the animals in the group have died”; when “the fraction of mice alive at the 90^th^ percentile survival age” was computed, the Long Rank (P<0.21) and the Wilcoxon (P<0.30) tests were not significant either.

**Table 3S. Statistical and analysis of total and partial survival curves in control and life-long atenolol-treated mice**

Chi square P<

Total curve

Long rank test 1.94 0.16

Wilcoxon test 1.10 0.29

Survival between 1,000 and 1,275 days

Log rank test 7.45 0.0063^b**^

Wilcoxon test: 6.70 P<0.0096^b**^

b* = significant difference in survival between control and life-long atenolol-treated animals.

**Table 4S. Heart rate and blood pressures in Old control and Old atenolol treated mice at 35 moths of age**

Old Old-AT

Heart rate (beats/min) 655.2±7.3 521.9±19.0^b***^

Arterial blood pressure (Systolic) 249.8±6.0 229.7±9.3^b*^

Arterial blood pressure (Mean) 213.4±7.7 190.6±9.6^b*^

Arterial blood pressure (Diastolic) 196.7±8.7 171.6±10.3^b*^

Values are means ± SEM from 8-16 different animals. Blood pressure units: mm of Hg. b* = significantly different from Old control; * P<0.05; *** P<0.001.

**Table 5S. Mitochondrial oxygen consumption in Young control, Old control, and Old atenolol treated mice**

Young Old Old-AT

**Heart:**

Glutamate/malate (state4) 65.9±.6.7 70.4±8.0 56.5±5.3

Glutamate/malate (state 3) 131.9±19.5 214.9±28.8^a*^ 143.8±13.8^b*^

Succinate+rotenone (state4) 101.3±6.7 100.2±10.7 95.5±6.6

Succinate+rotenone (state 3) 215.8±.26.8 270.4±.21.2 229.5±.25.5

**Skeletal muscle:**

Glutamate/malate (state4) 53.4±6.2 45.5±6.8 48.0±3.5

Glutamate/malate (state 3) 140.4±.24.2 147.9±19.8 134.8±26.0

Succinate+rotenone (state4) 61.6±11.0 62.3±5.5 61.1±6.8

Succinate+rotenone (state 3) 125.9±18.1 158.3±20.2 125.0±13.3

Values are means ± SEM from 5-8 different animals and are expressed as nanomoles of O_2_/min . mg protein. Old animals had 18 months of age. State 4: oxygen consumption in the absence of ADP. State 3: oxygen consumption in the presence of 500µM ADP a* = significantly different from Young control; b* = significantly different from Old control; * P<0.05.

**Table 6S. Mitochondrial ROS production in Young control, Old control, and Old atenolol treated mice**

Young Old Old-AT

**Heart:**

Glutamate/malate 0.33±0.04 0.26±0.05 0.26±0.05

Succinate+rotenone 1.08±0.21 1.02±0.15 0.99±0.16

Glutamate/malate+rotenone 1.30±0.12 1.67±0.14^a*^ 1.40±0.11

**Skeletal muscle:**

Glutamate/malate 0.41±0.02 0.42±0.03 0.34±0.03^a*^

Succinate+rotenone 0.88±0.12 0.88±0.08 0.83±0.09

Succinate 2.44±0.18 1.97±0.35 2.07±0.14^a*^

Glutamate/malate+rotenone 1.40±0.08 1.49±0.18 1.13±0.08^a*,b*^

Values are means ± SEM from 7-8 different animals except for succinate+rotenone in muscle (5-7 animals) and are expressed as nanomoles of H_2_O_2_/min . mg protein. a* = significantly different from Young control; b* = significantly different from Old control: * P<0.05.

**Table 7S. Amounts of respiratory complexes and AIF in mitochondria from Young control, Old control, and Old atenolol treated mice**

Young Old Old-AT

**Heart:**

Complex I 100±4..2 109.8±10.0 97.9±11.8

(39KDa subunit, NDUFA9)

Complex I 100±14.9 129.2±5.7 167.1±19.9^a**^

(30KDa subunit, NDUFS3)

Complex II 100±7.0 104.0±5.8 135.6±12.6^a*,b*^

(70 KDa subunit, Flavoprotein)

Complex III (48.5 KDa, CORE II) 100±4.6 121.6±15.8 115.3±11.1

Complex III (29.6 KDa subunit, 100±7.4 116.8±4.8 124.4±13.8

iron-sulfur protein)

Complex IV (57KDa subunit, COX I) 100±10.5 88.4±4.8 99.3±11.5

AIF 100±4.9 106.5±6.8 103.7±11.0

**Skeletal muscle:**

Complex I 100±10.4 100.8±15.1 130.9±16.3

(39KDa subunit, NDUFA9)

Complex I 100±14.8 79.5±7.7 77.7±11.4

(30KDa subunit, NDUFS3)

Complex II 100±10.6 58.7±3.8^a**^ 66.9±5.5^a**^

(70 KDa subunit, Flavoprotein)

Complex III (48.5 KDa, CORE II) 100±10.5 100.8±15.2 130.9±16.3

Complex III (29.6 KDa subunit, 100±9.9 150.0±8..2^a**^ 177.8±9.7^a***^

iron-sulfur protein)

Complex IV (57KDa subunit, COX I) 100±8.9 62.8±2.3^a**^ 54.7±4.9^a***^

AIF 100±5.8 96.1±10.6 75.8±6.9

Values are means ± SEM from 4 different animals. Units: ratio of complex I, II, III or IV and AIF/porin in arbitrary units. a* = significantly different from Young control; b* = significantly different from Old control; * P<0.05; ** P<0.01.

**Table 8S. Fatty acid composition (mol %) of heart mitochondrial lipids of Young control, Old control, and O**ld atenolol treated mice

| **Fatty acid** | **Young** | **Old** | **Old-AT** |
| --- | --- | --- | --- |
| **14:0** | 1.09±0.13 | 1.63±0.31 | 1.06±0.17^b*^ |
| **16:0** | 20.47±1.04 | 20.27±0.51 | 18.84±0.90 |
| **16:1n-7** | 2.67±0.62 | 1.39±0.15^a*^ | 0.96±0.06^a**^ |
| **18:0** | 15.63±1.26 | 18.64±0.69^a*^ | 18.50±0.68^a*^ |
| **18:1n-9** | 19.46±0.65 | 12.85±0.76^a***^ | 20.00±0.97^b***^ |
| **18:2n-6** | 7.47±0.69 | 6.23±0.43 | 7.90±1.10 |
| **18:3n-3** | 0.41±0.08 | 0.31±0.05 | 0.28±0.04 |
| **18:4n-3** | 0.77±0.19 | 0.67±0.13 | 0.60±0.13 |
| **20:0** | 0.52±0.16 | 0.28±0.03 | 0.33±0.03 |
| **20:1n-9** | 0.66±0.11 | 0.50±0.06 | 0.56±0.04 |
| **20:2n-6** | 0.52±0.06 | 0.59±0.08 | 0.48±0.06 |
| **20:3n-6** | 0.34±0.03 | 1.61±0.19^a***^ | 0.28±0.02^b***^ |
| **20:4n-6** | 4.33±0.42 | 3.17±0.31^a*^ | 4.68±0.06^b**^ |
| **20:5n-3** | 0.36±0.06 | 0.19±0.02^a*^ | 0.19±0.02^a**^ |
| **22:0** | 0.34±0.01 | 0.22±0.03^a*^ | 0.23±0.03^a*^ |
| **22:4n-6** | 0.40±0.05 | 0.31±0.02^a*^ | 0.32±0.01 |
| **22:5n-6** | 0.50±0.03 | 0.41±0.03 | 0.47±0.04 |
| **22:5n-3** | 1.90±0.33 | 1.73±0.14 | 1.67±0.18 |
| **24:0** | 1.33±0.30 | 0.56±0.06^a**^ | 0.69±0.06^a*^ |
| **22:6n-3** | 19.92±0.77 | 28.03±1.01^a***^ | 21.56±1.74^b**^ |
| **24:5n-3** | 0.53±0.05 | 0.18±0.04^a***^ | 0.41±0.04^a*,b**^ |
| **24:6n-3** | 0.26±0.03 | 0.15±0.01^a*^ | 0.21±0.02 |

Values are means ± SEM from 5-6 different animals. For abbreviations see the Materials and Methods section. a* = significantly different from Young control; b* = significantly different from Old control;; * P<0.05; ** P<0.01; *** P<0.001.

**Table 9S. Fatty acid composition (mol %) of skeletal muscle mitochondrial lipids of Young control, Old control, and Old atenolol treated** mice

| **Fatty acid** | **Young** | **Old** | **Old-Atenolol** |
| --- | --- | --- | --- |
| **14:0** | 1.10±0.08 | 1.32±0.21 | 1.43±0.11 |
| **16:0** | 22.03±0.86 | 22.94±0.34 | 25.23±0.97^a*^ |
| **16:1n-7** | 2.71±0.08 | 2.87±0.13 | 3.00±0.29 |
| **18:0** | 13.70±0.57 | 14.16±0.82 | 15.58±1.74 |
| **18:1n-9** | 19.52±1.08 | 16.39±0.47^a*^ | 21.27±1.44^b**^ |
| **18:2n-6** | 12.37±0.71 | 10.54±0.82 | 9.27±1.33^a*^ |
| **18:3n-3** | 0.42±0.06 | 0.43±0.03 | 0.60±0.15 |
| **18:4n-3** | 0.57±0.10 | 0.66±0.10 | 0.67±0.11 |
| **20:0** | 0.26±0.02 | 0.29±0.06 | 0.31±0.04 |
| **20:1n-9** | 0.52±0.13 | 0.46±0.04 | 0.42±0.02 |
| **20:2n-6** | 0.65±0.04 | 0.60±0.12 | 0.92±0.12^b*^ |
| **20:3n-6** | 1.33±0.04 | 1.37±0.17 | 1.77±0.26 |
| **20:4n-6** | 4.11±0.31 | 2.53±0.17^a***^ | 2.58±0.19^a***^ |
| **20:5n-3** | 0.23±0.01 | 0.23±0.03 | 0.20±0.03 |
| **22:0** | 0.24±0.03 | 0.20±0.01 | 0.21±0.04 |
| **22:4n-6** | 0.39±0.04 | 0.35±0.05 | 0.30±0.02 |
| **22:5n-6** | 0.46±0.05 | 0.42±0.04 | 0.36±0.03 |
| **22:5n-3** | 2.31±0.10 | 1.77±0.17^a*^ | 1.56±0.11 ^a***^ |
| **24:0** | 0.69±0.09 | 0.67±0.11 | 0.66±0.08 |
| **22:6n-3** | 15.62±0.48 | 21.29±0.53^a***^ | 13.15±1.12^a*,b***^ |
| **24:5n-3** | 0.37±0.08 | 0.21±0.02^a*^ | 0.19±0.03^a*^ |
| **24:6n-3** | 0.27±0.05 | 0.21±0.01 | 0.21±0.02 |

Values are means ± SEM from 6 different animals. For abbreviations see the Materials and Methods section. a* = significantly different from Young control; b* = significantly different from Old control; * P<0.05; ** P<0.01; *** P<0.001.

**Table 10S. Oxidative damage in mitochondrial DNA in Young control, Old control, and Old atenolol treated mice.**

Young Old Old-AT

Heart 15.31±3.40 18.88±3.95 10.86±1.45^b*^

Skeletal muscle 3.59±1.02 7.06±2.19 7.20±1.72

Values are means ± SEM from 5-7 different animals except for muscle of Young controls (n=5) and are expressed as 8-oxodG/10^5^dG. b* = significantly different from Old control; * P<0.05


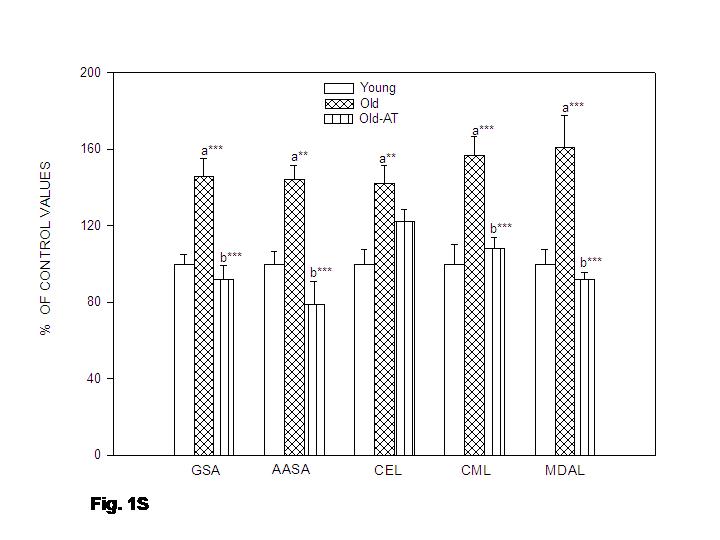


**Fig. 1S.** Protein oxidation, glycoxidation and lipoxidation indicators in skeletal muscle mitochondria from Young control, Old control and Old atenolol treated mice. Values are means ± SEM from 6 different animals and are expressed as percentage of those in the Young controls for each protein modification marker. Control values: 3,029±152 (glutamic semialdehyde, GSA); 322±21 (AASA, aminoadipic semialdehyde, AASA); 268±20 (carboxyethyl-lysine, CEL); 491±50 (carboxymethyl-lysine, CML); 392±29 (malondialdehyde-lysine, MDAL). Units: µmol/mol lysine. a*: significantly different from Young controls; b*: significantly different from Old controls; ** P<0.01; *** P<0.001.

**Detailed Methods**

*Animals and study design*

A total of 134 B6D2F1 (C57BL/6 female x DBA/2 male**)** male mice were used and were long-term maintained in a separate room of the animal house during their whole lifespan or until sacrificed at young or old age. 86 of these animals (43 Old control and 43 Old-AT-treated) were used only to obtain the survival curves. These animals were maintained under optimum conditions [12:12 (light-dark) cycle, 22ºC ± 2ºC and 50% ± 10% relative humidity, in individual mouse cages], were left intact during their whole lifespan (except for the AT-treatment) and their day of spontaneous death was recorded. The treatment with atenolol was started at 2 months of age. A separated group of 48 “pilot” animals (24 controls and 24 AT-treated) was established at the beginning of the experiment in order to measure the different physiological and biochemical parameters when reaching old age (after 16 months of experimentation). Among the 24 animals of each of these two groups (control and AT-treated), 8 animals were used for the ROS and oxygen consumption measurements in isolated mitochondria, 8 animals for the measurement of 8-oxodG, and 8 animals to assay the rest of the biochemical parameters after being sacrificed by cervical dislocation. At the time of sacrifice these animals had 18 months of age. A young control group (6 months of age-Young control) was included using mice that were born 6 months before the sacrifice of the 18 months old pilot animals and were maintained in parallel in the same room and conditions than the rest of the animals. The animals in the atenolol group had free access to a solution of 0.1 g/L of atenolol (Sigma, A7655) in drinking water, which resulted in a mean atenolol intake of 0.559 mg per mouse per day. The diet (Panlab, Spain) was offered *ad libitum* to all animals. Just after cervical dislocation, hearts and skeletal muscle (total hind limb muscle) were immediately processed to isolate functional mitochondria, which were used to measure mitochondrial respiration and rates of ROS generation. Whole hearts, spleen and muscle tissue samples were stored at -80ºC for the posterior analyses of the rest of the biochemical parameters. All the experiments in were approved by the Experimental Animal Committee from the Complutense University.

*Physiological parameters*

Rectal temperature was measured using a rectal probe (Thermocouple thermometer model 8112-20, Cole-Parmer Instrument Company). The measurements were performed three times in each mouse, always at 11:00, on three different days separated 15 days from each other, during the last 2 months of experimentation. To estimate the routine metabolic rate, individual mice were placed inside a closed-system respirometer (total volume 2,600 ml) and the carbon dioxide produced was captured with a 10% KCl solution. The rate of oxygen consumption of each animal was measured at rest with an oxygen analyzer and probe (Model 600 Can 1691, Engineered Systems & Designs) at 23±1°C. Heart rate and blood pressures were measured in conscious mice with a noninvasive tail-cuff manometry system (LE5001 Panlab Harvard Apparatus). Each animal was acclimatized for at least three practice sessions in the three consecutive weeks before the final measurements were recorded. In each session 8 consecutive readings were recorded and their average was used to obtain systolic, diastolic, and mean blood pressure. These measurements were performed during the last 2 months of experimentation.

*Isolation of functional mitochondria*

Mitochondria were obtained from fresh tissue by the procedure of Mela and Seitz^1^ with modifications. One whole heart or 60 grams of muscle tissue were chopped into small pieces and were homogenized with a loose-fitting glass-glass pestle in 10 ml of isolation buffer (220 mM mannitol, 70 mM sucrose, 1 mM EDTA, 10 mM Tris-HCl, pH 7.4) containing 5 mg of protease (subtilisin A Type VIII) and 25 mg of fatty acid-free albumin. After the samples stood for 1 min, 25 ml of additional isolation buffer containing 25 mg of albumin were added, and gentle homogenization was performed again with a tighter fitting pestle. The nuclei and cell debris were removed by centrifugation at 700 x g for 10 min. Mitochondria were obtained by centrifuging the supernatant at 8,000 x g for 10 min. The mitochondrial pellets were resuspended in 250 µl of isolation buffer. All the above procedures were performed at 4ºC. Mitochondrial protein was measured by the biuret method. The final mitochondrial suspensions were maintained at high concentration of mitochondrial protein over ice and were immediately used for the oxygen consumption and H_2_O_2_ production measurements during the next two hours.

*Mitochondrial oxygen consumption*

The rate of oxygen consumption of heart and skeletal muscle mitochondria was measured at 37ºC in a water-thermostatized incubation chamber with a computer-controlled Clark-type O2 electrode (Oxygraph, Hansatech, UK) in 0.5 ml of incubation buffer (145 mM KCl, 30 mM Hepes, 5 mM KH2PO4, 3 mM MgCl2, 0.1 mM EGTA, 0.1% albumin, pH 7.4). The substrates used were complex I-linked (2.5 mM glutamate/2.5 mM malate) or complex II-linked (5 mM succinate+rotenone). The assays were performed in the absence (State 4-resting) and in the presence (State 3-phosphorylating) of 500 µM ADP.

*Mitochondrial H_2_O_2_ generation*

The rate of mitochondrial ROS production (O_2_^.-^ + H_2_O_2_) was assayed by measuring the increase in fluorescence as a function of time (excitation at 312 nm, emission at 420 nm) due to oxidation of homovanillic acid by H_2_O_2_ in the presence of horseradish peroxidase, essentially as described^2,3^. Reaction conditions were 0.25 mg of mitochondrial protein per ml, 6 U/ml of horseradish peroxidase, 0.1 mM homovanillic acid, 50 U/ml of superoxide dismutase, and 2.5 mM glutamate/2.5 mM malate, or 5 mM succinate with (to block backward electron flow to complex I) and without 2 µM rotenone as substrates, added at the end (to start the reaction) to the incubation buffer (145 mM KCl, 30 mM Hepes, 5 mM KH_2_PO_4_, 3 mM MgCl_2_, 0.1 mM EGTA, 0.1% albumin, pH 7.4) at 37ºC, in a total volume of 1.5 ml. Additional experiments with glutamate/malate+2µM rotenone were performed to assay maximum rates of complex I ROS generation. Duplicated samples were incubated for 15 min at 37º C. The reaction was stopped by transferring the samples to a cold bath and adding 0.5 ml of stop solution (2.0 M glycine, 2.2 M NaOH, 50 mM EDTA, pH 12), and the fluorescence was read in a LS50B Perkin-Elmer fluorometer. Known amounts of H_2_O_2_ generated in parallel by glucose oxidase with glucose as substrate were used as standards. Since the superoxide dismutase added in excess converts all O_2_^.-^ excreted by mitochondria (if any) to H_2_O_2_, the measurements represent the total (O_2_^.-^ plus H_2_O_2_) rate of mitochondrial ROS production.

*Measurement of mitochondrial complexes I to IV, AIF, and ERK*

The amounts of a) the mitochondrial respiratory chain complexes (I to IV), b) the complex I regulatory factor AIF, and c) the ERK1/2 pathway (ERK1/2 and phospho-ERK1/2) were estimated using western blot analyses. Samples were homogenized in a buffer containing 180 mM KCl, 5 mM MOPS, 2 mM EDTA, 1 mM diethylenetriaminepentaacetic acid, 1 µM butylated hydroxyltoluene, protease inhibitor mix (80-6501-23, Amersham Biosciences) and phosphatase inhibitors (Na_3_VO_4_ 1 mM, NaF 1mM). After a brief centrifugation (1000 rpm for 3 min at 4°C) to pellet cellular debris, proteins concentrations were measured in the supernatants using the Bradford method (Bio-Rad Protein Assay 500-0006). Proteins were separated by one-dimensional SDS-PAGE. Samples were mixed with sample buffer (62.5 mM Tris–HCl pH 6.8, 2% SDS, 10% glycerol, 20% 2-β-mercaptoethanol and 0.02% bromophenol blue) and heated for 5 min at 95°C. Proteins (10 μg for the respiratory chain complexes and AIF, and 80 µg for the others) were subjected to electrophoresis on 10% SDS-polyacrylamide minigels. For immunodetection, proteins were transferred using a Mini Trans-Blot Transfer Cell (Bio Rad) in a buffer containing 25 mM TRIS, 192 mM Glycine and 20% methanol, to polyvinylidene difluoride membranes (Immobilon-P Millipore, Bedford, MA). The membranes were immersed in blocking solution (0.2% I-Block Tropix AI300, 0.1% Tween in PBS) for 1 h at room temperature. After blocking, the membrane was washed two times using 0.05% TBS-T buffer. Afterwards, the membrane was incubated in primary solution using specific antibodies for the 40kDa (NDUFA9) and 30kDa (NDUFS3) subunits of complex I (1:1000 in both cases; ref. A21344 and A21343, respectively; Molecular Probes), 70kDa subunit (Flavoprotein) of complex II (1:500; ref. A11142, Molecular Probes), 50kDa (CORE 2) and 30kDa (Rieske iron-sulfur protein) subunits of complex III (1:1000 in both cases; ref. A11143 and A21346, respectively; Molecular Probes), 40kDa COXI subunit of complex IV (1:1000; ref. A6403; Molecular Probes), anti-AIF (1:1000, ref. A7549, Sigma), anti-phospho-ERK1/2 (1:500, ref. 4370, Cell Signaling Technology), and anti-ERK1/2 (1:500, ref. 4695, Cell Signaling Technology). Anti-phospho-ERK1/2 and anti-ERK1/2 were incubated in the same membrane after stripping of the first antibody. An antibody to porin (1:15000, ref. A31855, Molecular Probes) and to tubulin ( 1:5000 , ref. ab7291, Abcam) was also used in order to determine the proportion of protein levels referred to total mitochondrial and total mass, respectively. The primary antibody was incubated 1hour at room temperature, except ERK1/2 and ERK1/2 which were incubated 16 hr at 4°C. The membrane was washed three times in 0.05% TBS-T buffer and incubated 1 hour at room temperature with the appropriate secondary antibodies [ECL Anti-mouse IgG, horseradish Peroxidase linked whole antibody-NA93IV GE Healthcare (1:5000) and ImmunoPure Goat Anti-Rabbit IgG, (H+L), peroxidase conjugated- 31460 Pierce Biotechnology (1:100000)]. After five washes with 0.05% TBS-T buffer, bands were visualized using an enhanced chemiluminescence HRP substrate (Millipore, MA, USA). Signal quantification and recording was performed with a ChemiDoc equipment (Bio-Rad Laboratories, Inc., Barcelona, Spain).

*Fatty acid analyses and their biosynthetic enzymes, and global fatty acid unsaturation indexes*

Fatty acids from mitochondrial lipids were analyzed as methyl esters derivatives by gas chromatography (GC) as previously described.^4^ Separation was performed with a DBWAX capillary column (30 m x 0.25 mm x 0.20 μm) in a GC System 7890A with a Series Injector 7683B and a FID detector (Agilent Technologies, Barcelona, Spain). Identification of fatty acid methyl esters was made by comparison with authentic standards (Larodan Fine Chemicals, Malmö, Sweden). Results are expressed as mol%. The density of double bonds in the membrane was calculated by the Double Bond Index, DBI = [(1×Σmol% monoenoic) + (2×Σmol% dienoic) + (3×Σmol% trienoic) + (4×Σmol% tetraenoic) + (5×Σmol% pentaenoic) + (6×Σmol% hexaenoic)]. The membrane susceptibility to lipid peroxidation was calculated by the Peroxidizability Index, PI= [(0.025×Σmol% monoenoic) + (1×Σmol% dienoic) + (2×Σmol% trienoic) + (4×Σmol% tetraenoic) + (6×Σmol% pentaenoic) + (8×Σmol% hexaenoic)].

*Oxidative damage to mtDNA (8-oxodG)*

Isolation of mtDNA was performed by the method of Latorre and cols.^5^ adapted to mammals.^6^ The isolated mitochondrial DNA was digested to deoxynucleoside level by incubation at 50ºC with 5 U of nuclease P1 (in 20 µl of 20 mM sodium acetate, 10 mM ZnCl2, 15% glycerol, pH 4.8) for 30 min and 1 U of alkaline phosphatase (in 20 µL of 1 M Tris-HCl, pH 8.0) for 1 hr**.** All aqueous solutions used for mtDNA isolation, digestion and chromatographic separation were prepared in HPLC-grade water. Steady-state oxidative damage to mtDNA was estimated by measuring the level of 8-oxo-7,8-dihydro-2’deoxyguanosine (8-oxodG) referred to that of the non-oxidized base (deoxyguanosine, dG). 8-oxodG and dG were analyzed by HPLC with on line electrochemical and ultraviolet detection respectively. The nucleoside mixture was injected into a reverse-phase Mediterranea Sea 18 column (5µm, 4.6 mm x 25 cm; Teknokroma, Barcelona, Spain), and was eluted with a mobile phase containing 6.5% acetonitrile and 50 mM phosphate buffer pH 5.0. The volume of sample injected in the column was 100 µl. A Gilson 305 pump with nanometric module 805 at 0.9 ml/min was used. 8-oxodG was detected with an ESA Coulochem II electrochemical coulometric detector (ESA, Inc. Bedford, MA) with a 5011A analytical cell run in the oxidative mode (350 mV/ 20 nA), and dG was detected with a Biorad model 1806 UV detector at 254 nm. For quantification, peak areas of dG standards and of three level calibration pure 8-oxodG standards (Sigma) were analyzed during each HPLC run. Comparison of areas of 8-oxodG standards injected with and without simultaneous injection of dG standards ensured that no oxidation of dG occurred during the chromatography.

*Oxidation-derived protein damage markers*

GSA, AASA, CML, CEL and MDAL were determined as trifluoroacetic acid methyl esters (TFAME) derivatives in acid hydrolyzed delipidated and reduced mitochondrial protein samples by GC/MS^8^ using a HP6890 Series II gas chromatograph (Agilent, Barcelona, Spain) with a MSD5973A Series detector and a 7683 Series automatic injector, a HP-5MS column (30-m x 0.25-mm x 0.25-µm), and the described temperature program.^7^ Quantification was performed by internal and external standardization using standard curves constructed from mixtures of deuterated and non-deuterated standards. Analyses were carried out by selected ion-monitoring GC/MS (SIM-GC/MS). The ions used were: lysine and [^2^H_8_]lysine, *m/z* 180 and 187, respectively; 5-hydroxy-2-aminovaleric acid and [^2^H_5_]5-hydroxy-2-aminovaleric acid (stable derivatives of GSA), *m/z* 280 and 285, respectively; 6-hydroxy-2-aminocaproic acid and [^2^H_4_]6-hydroxy-2-aminocaproic acid (stable derivatives of AASA), *m/z* 294 and 298, respectively; CML and [^2^H_4_]CML, *m/z* 392 and 396, respectively; CEL and [^2^H_4_]CEL, *m/z* 379 and 383, respectively; and MDAL and [^2^H_8_]MDAL, *m/z* 474 and 482, respectively. The amounts of product were expressed as µmoles of GSA, AASA, CML, CEL or MDAL per mol of lysine.

*Immune function parameters*

Spleens were removed aseptically, freed of fat, minced with scissors and gently pressed through a mesh screen (Sigma, St Louis, and USA). The cell suspensions were centrifuged in a gradient of Ficoll-Hypaque (Sigma) with a density of 1.070 g/ml. Cells from the interface were re-suspended in RPMI 1640 medium enriched with L-glutamine (PAA, Pasching Austria) and supplemented with 10% heat-inactivated fetal calf serum (Gibco, Canada) and 1% gentamicin (10 mg/ml, Gibco). They were then washed, and the number of leukocytes was determined. Cellular viability, routinely measured before and after each experiment by the trypan-blue exclusion test, was higher than 95% in all cases. All incubations were performed at 37 °C in a humidified atmosphere of 5% CO_2_. Lymphoprolipheration, chemotaxis (directed migration) and Natural killer (NK) activity assays were performed as previously described.^8^

*Behavioral tests*

The experiments were performed from 08:30 to 13:00 h in accordance with the 53/2013 Spanish legislation on “Protection of Animals Used for Experimental and Other Scientific Purposes” and the European Directive 2010/63/EU from the European Parliament on this subject. Behavioral testing took place during four consecutive days. On the first day, animals were subjected to the sensorimotor tests (wood rod test and tightrope test). Then, the tests to analyze exploratory and anxiety-like behaviors (the open field and holeboard tests) were carried out. The sequence of testing was based on previous reports by different authors.^9,10^ All those behavioral tests were performed as previously described.^11^ Behavior was evaluated by three independent observers. Olfactory trails were removed by cleaning the surfaces of the apparatuses after each test.

*Statistics*

Comparisons between the three groups of animals were performed by One-Way ANOVA. Comparison of long-life survival of Old control and Old AT-treated animals was analyzed with the Log Rank and Wicoxon tests. P<0.05 was selected as the minimum level of statistical significance.

**Supplemental references**

1. Mela L, Seitz S. Isolation of mitochondria with emphasis on heart mitochondria from small amounts of tissue. *Methods Enzymol.* 1997; 55:39-46.
2. Barja G. The quantitative measurement of H_2_O_2_ generation in isolated mitochondria. *J Bioenerg Biomembr.* 2002: 34:227-233.
3. Sanz A, Barja G. Estimation of the rate of production of oxygen radicals by mitochondria. In: Conn M, ed. *Handbook of Models for Human Aging*, New York: Academic Press; 2006: 183-189.
4. Sanchez-Roman I, Gomez J, Naudi A, Ayala V, Portero-Otín M, Lopez-Torres M, Pamplona R, Barja G (2010) The β-blocker atenolol lowers the longevity-related degree of fatty acid unsaturation, decreases protein oxidative damage and increases ERK signaling in the heart of C57BL/6 mice. *Rejuv. Res.* 13, 683-693.
5. Latorre A, Moya A, Ayala A. Evolution of mitochondrial DNA in *Drosophila suboscura*. *PNAS* 1986; 83:8649-8653.
6. Asunción JG, Millan A, Pla R, Bruseghini L, Esteras A, Pallardo FV, Sastre J, Viña J. Mitochondrial glutathione oxidation correlates with age-associated oxidative damage to mitochondrial DNA. *FASEB J* 1996; 10:333-338.
7. Pamplona R, Dalfó E, Ayala V, Bellmunt MJ, Ferrer I, Portero-Otín M. Proteins in human brain cortex are modified by oxidation, glycoxidation and lipoxidation. Effects of Alzheimer disease and identification of lipoxidation targets. *J Biol Chem.* 2005; 280: 21522-21530.
8. Arranz L, De Castro N.M., Baeza I, Gimenez-Llort L, De la Fuente M. Effect of environmental enrichment on the immuneendocrine afing of male and female triple-transgenic 3xTg-AD mice for Alzheimer´s disease. *J Alzheimer´s Disease* 2011; 25:727-737.
9. Johansson B, [Halldner L](http://www.ncbi.nlm.nih.gov/sites/entrez?Db=pubmed&Cmd=Search&Term="Halldner L"%5BAuthor%5D&itool=EntrezSystem2.PEntrez.Pubmed.Pubmed_ResultsPanel.Pubmed_DiscoveryPanel.Pubmed_RVAbstractPlus), [Dunwiddie TV](http://www.ncbi.nlm.nih.gov/sites/entrez?Db=pubmed&Cmd=Search&Term="Dunwiddie TV"%5BAuthor%5D&itool=EntrezSystem2.PEntrez.Pubmed.Pubmed_ResultsPanel.Pubmed_DiscoveryPanel.Pubmed_RVAbstractPlus), [Masino SA](http://www.ncbi.nlm.nih.gov/sites/entrez?Db=pubmed&Cmd=Search&Term="Masino SA"%5BAuthor%5D&itool=EntrezSystem2.PEntrez.Pubmed.Pubmed_ResultsPanel.Pubmed_DiscoveryPanel.Pubmed_RVAbstractPlus), [Poelchen W](http://www.ncbi.nlm.nih.gov/sites/entrez?Db=pubmed&Cmd=Search&Term="Poelchen W"%5BAuthor%5D&itool=EntrezSystem2.PEntrez.Pubmed.Pubmed_ResultsPanel.Pubmed_DiscoveryPanel.Pubmed_RVAbstractPlus), [Giménez-Llort L](http://www.ncbi.nlm.nih.gov/sites/entrez?Db=pubmed&Cmd=Search&Term="Giménez-Llort L"%5BAuthor%5D&itool=EntrezSystem2.PEntrez.Pubmed.Pubmed_ResultsPanel.Pubmed_DiscoveryPanel.Pubmed_RVAbstractPlus), [Escorihuela RM](http://www.ncbi.nlm.nih.gov/sites/entrez?Db=pubmed&Cmd=Search&Term="Escorihuela RM"%5BAuthor%5D&itool=EntrezSystem2.PEntrez.Pubmed.Pubmed_ResultsPanel.Pubmed_DiscoveryPanel.Pubmed_RVAbstractPlus), [Fernández-Teruel A](http://www.ncbi.nlm.nih.gov/sites/entrez?Db=pubmed&Cmd=Search&Term="Fernández-Teruel A"%5BAuthor%5D&itool=EntrezSystem2.PEntrez.Pubmed.Pubmed_ResultsPanel.Pubmed_DiscoveryPanel.Pubmed_RVAbstractPlus), [Wiesenfeld-Hallin Z](http://www.ncbi.nlm.nih.gov/sites/entrez?Db=pubmed&Cmd=Search&Term="Wiesenfeld-Hallin Z"%5BAuthor%5D&itool=EntrezSystem2.PEntrez.Pubmed.Pubmed_ResultsPanel.Pubmed_DiscoveryPanel.Pubmed_RVAbstractPlus), [Xu XJ](http://www.ncbi.nlm.nih.gov/sites/entrez?Db=pubmed&Cmd=Search&Term="Xu XJ"%5BAuthor%5D&itool=EntrezSystem2.PEntrez.Pubmed.Pubmed_ResultsPanel.Pubmed_DiscoveryPanel.Pubmed_RVAbstractPlus), [Hårdemark A](http://www.ncbi.nlm.nih.gov/sites/entrez?Db=pubmed&Cmd=Search&Term="Hårdemark A"%5BAuthor%5D&itool=EntrezSystem2.PEntrez.Pubmed.Pubmed_ResultsPanel.Pubmed_DiscoveryPanel.Pubmed_RVAbstractPlus), [Betsholtz C](http://www.ncbi.nlm.nih.gov/sites/entrez?Db=pubmed&Cmd=Search&Term="Betsholtz C"%5BAuthor%5D&itool=EntrezSystem2.PEntrez.Pubmed.Pubmed_ResultsPanel.Pubmed_DiscoveryPanel.Pubmed_RVAbstractPlus), [Herlenius E](http://www.ncbi.nlm.nih.gov/sites/entrez?Db=pubmed&Cmd=Search&Term="Herlenius E"%5BAuthor%5D&itool=EntrezSystem2.PEntrez.Pubmed.Pubmed_ResultsPanel.Pubmed_DiscoveryPanel.Pubmed_RVAbstractPlus), [Fredholm BB](http://www.ncbi.nlm.nih.gov/sites/entrez?Db=pubmed&Cmd=Search&Term="Fredholm BB"%5BAuthor%5D&itool=EntrezSystem2.PEntrez.Pubmed.Pubmed_ResultsPanel.Pubmed_DiscoveryPanel.Pubmed_RVAbstractPlus). Hyperalgesia, anxiety, and decreased hypoxic neuroprotection in mice lacking the adenosine A1 receptor. [*PNAS*.](javascript:AL_get(this, 'jour', 'Proc Natl Acad Sci U S A.');) 2001; 98:9407-9412.
10. Giménez-Llort L, Fernández-Teruel A, Escorihuela RM, Fredholm BB, Tobeña A, Pekín M, Johansson B, 2002. Mice lacking the adenosine A1 receptor are anxious and aggressive, but are normal learners with reduced muscle strength and survival rate. *Eur J Neurosci*. 2002; 16: 547-550.
11. Baeza I, De Castro NM, Gimenez-Llort L, De la Fuente M. Ovariectomy, a model of menopause in rodents, causes a premature aging of the nervous and immune systems. *J Neuroimmunol.* 2010; 219:90-99.

Raw data tables in Excel “RAW DATA TABLE OF FIGURE 1 Kaplan-Meyer survival plot CONTROL AND ATENOLOL.xls” corresponding to survival curves in Fig. 1 (Kaplan-Meyer survival plots) during the whole life span of control and chronically atenolol-treated animals:

1.Cohort: Control animals

2. Cohort: Atenolol-treated animals
